# A geometric framework for understanding dynamic information integration in context-dependent computation

**DOI:** 10.1016/j.isci.2021.102919

**Published:** 2021-07-30

**Authors:** Xiaohan Zhang, Shenquan Liu, Zhe Sage Chen

**Affiliations:** 1 School of Mathematics, South China University of Technology, Guangzhou, China; 2 Department of Psychiatry, Department of Neuroscience and Physiology, Neuroscience Institute, New York University Grossman School of Medicine, New York City, NY, USA

**Keywords:** Neuroscience, Cognitive Neuroscience, Biocomputational Method

## Abstract

The prefrontal cortex (PFC) plays a prominent role in performing flexible cognitive functions and working memory, yet the underlying computational principle remains poorly understood. Here, we trained a rate-based recurrent neural network (RNN) to explore how the context rules are encoded, maintained across seconds-long mnemonic delay, and subsequently used in a context-dependent decision-making task. The trained networks replicated key experimentally observed features in the PFC of rodent and monkey experiments, such as mixed selectivity, neuronal sequential activity, and rotation dynamics. To uncover the high-dimensional neural dynamical system, we further proposed a geometric framework to quantify and visualize population coding and sensory integration in a temporally defined manner. We employed dynamic epoch-wise principal component analysis (PCA) to define multiple task-specific subspaces and task-related axes, and computed the angles between task-related axes and these subspaces. In low-dimensional neural representations, the trained RNN first encoded the context cues in a cue-specific subspace, and then maintained the cue information with a stable low-activity state persisting during the delay epoch, and further formed line attractors for sensor integration through low-dimensional neural trajectories to guide decision-making. We demonstrated via intensive computer simulations that the geometric manifolds encoding the context information were robust to varying degrees of weight perturbation in both space and time. Overall, our analysis framework provides clear geometric interpretations and quantification of information coding, maintenance, and integration, yielding new insight into the computational mechanisms of context-dependent computation.

## Highlights

• Units with mixed selectivity emerged in context-dependent computation
• Neural sequences emerged in the trained RNN during cue delay
• Task-specific neural trajectories distinguished in low-dimensional subspaces
• Sensory integration formed dynamic fixed points and line attractors

## Graphical abstract

**Figure:**
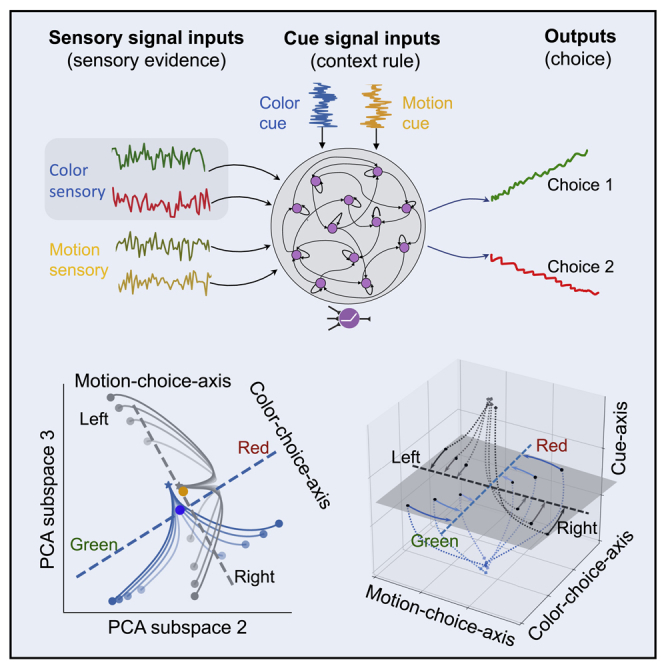

## Introduction

Cognitive flexibility is an important characteristic that enables animals or humans to selectively switch between sensory inputs to generate appropriate behavioral responses (Diamond, 2013; Scott, 1962; Miyake and Friedman, 2012). This important process has been associated with various goal-directed behaviors, including multi-tasking and decision-making (Thea, 2012; Dajani and Uddin, 2015; Le et al., 2018; Pezzulo et al., 2014). Impaired cognitive flexibility has been observed among individuals with mental illnesses, such as schizophrenia, (Woodward et al., 2012; Maud et al., 2012) and those at risk for mental disorders (Murphy et al., 2012; Chamberlain et al., 2007; Vaghi et al., 2017). Therefore, identifying the computational principle underlying cognitive flexibility may improve our understanding of brain dysfunction. The prefrontal cortex (PFC) is known to contribute to cognitive flexibility, serving as the main storage of temporary working memory (WM) to represent and maintain contextual information (Baddeley, 2003; Miller, 2000; Todd et al., 2009). Neurophysiological recordings have shown that single PFC cells respond selectively to different task-related parameters (White and Wise, 1999; Eiselt and Nieder, 2016; Hyman et al., 2013; Machens et al., 2010; Rigotti et al., 2013) and the activity of PFC pyramidal neurons can maintain WM to perform context-dependent computation (Wallis et al., 2001). However, due to the heterogeneity and diversity of single-neuron responses, it remains challenging to understand how task-modulated single-neuron activities integrate task-related information to guide subsequent decision-making. To address this knowledge gap, researchers relied on population coding to understand the maintenance and manipulation of context information in decision-making tasks (Meyers et al., 2008; Cichy et al., 2014; King and Dehaene, 2014; Lundqvist et al., 2016). In the literature, several computational theories and analysis methods have been proposed (Wu et al., 2020; Mante et al., 2013). However, how the sensory information is integrated via dynamic population coding in a context-dependent manner remains poorly understood. Meanwhile, an intuitive and interpretable dynamical systems framework for context-dependent WM and decision making is lacking.

**Figure 1:**
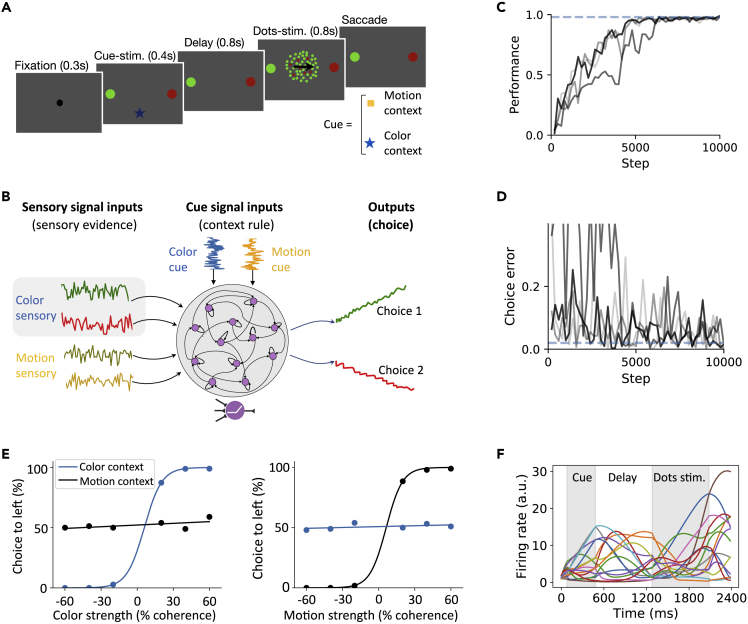
Trained RNN to perform the delayed context-dependent integration task (A) Behavioral task description. A monkey was trained to discriminate, depending on the contextual cue, either the predominant color or predominant motion direction of randomly moving dots, and to further indicate its decision with a saccadic eye movement to a choice target. The cue stimulus onset determined the current context, which was characterized by different shapes and colors of the fixation point. The cue stimulus was followed by a fixed-delay epoch, and then followed by randomly moving dots stimuli. The monkey was rewarded for a saccade to the target matching the current context. (B) Schematic of a fully connected, nonlinear RNN in context-dependent computation. The network received noisy inputs of two types: time-varying sensory stimulus and cue input. The stimulus inputs consisted of four task-relevant sensory information, each represented by an input unit that encoded the evidence for the direction of stimulus. The cue inputs consisted of two cue signals, which were represented by two input units (that indicated the current context and instructed the network to distinguish the type of stimulus). The two output channels encoded the response direction. (C and D) RNN learning curve (C) and the performance curve (D). Training was completed once both quantities reached the convergence criterion (blue horizontal dashed lines). (E) Psychometric curves in a delayed context-dependent integration task. The probability of a correct direction judgment is plotted as a function of color (*Left*) and motion (*Right*) coherence in color-context (blue) and motion-context (black) trials. (F) The activities of representative units indicated by different colors. The first gray shading area indicates cue stimulus epoch and the second shading area indicates the presentation of random dots (i.e., integration of sensory stimulus epoch)

Recurrent neural networks (RNNs) have been widely used for modeling a wide range of neural circuits, such as the PFC and parietal cortex, in performing various cognitive tasks (Rajan et al., 2016; Hennequin et al., 2014; Sussillo et al., 2015). However, computational mechanisms of RNNs in performing those tasks remain elusive because of the black box modeling. In this paper, we trained an RNN to perform a delayed context-dependent task (Figure 1A) and proposed a geometric analysis framework to understand dynamic population coding and information integration. We found that the trained RNN captured critical physiological properties consistent with reported experimental data. Additionally, the trained RNN showed some emergent features of neuronal activity observed in the PFC, such as the mixed selectivity and sequential activity. Based on dimensionality reduction of population responses, we defined task epoch-specific subspaces and dynamic attractors during the sensory integration epoch, and showed that the context-configured network state is temporally tuned to regulate sensory integration to guide decision-making. Together, our analysis framework not only helps uncover the computational mechanisms of encoding and maintenance of context information in decision-making but also helps illustrate information integration based on interpretable geometric concepts.

## Results

### Trained RNN for performing a delayed context-dependent integration task

We trained the RNN to perform a delayed context-dependent WM or decision-making task (Figure 1B). At each trial, the network received two types of noisy inputs: sensory stimulus and cue stimulus. The sensory input units encoded the momentary motion and color evidence toward two target directions. The cue input units encoded the contextual signal, instructing the network to discriminate a specific type of sensory input. The choice output units encoded the response direction. All units had non-negative and non-saturating firing rates to mimic the properties of biological neurons (Priebe and Ferster, 2008; Abbott and Chance, 2005).

Upon successful convergence of RNN training (Figures 1C and 1D), psychometric tests showed that the trained RNN captured critical physiological properties consistent with experimental findings (Figure 1E). For example, the trained network achieved better performance with higher color coherence stimulus in the color context, but not in the motion context; and vice versa (Figure 1E). Units in the trained RNN showed diverse firing rate profiles at different task epochs (Figure 1F). Furthermore, we analyzed the impact of the proportion of zero recurrent weights on the task performance (Figure S1A). The trained RNN exhibited a strong self-connection (Figure S1B). Additionally, recurrent weight perturbation analyses were also used to assess the stability of the trained RNN (Figure S2).

### Single Unit Responses

#### Mixed selectivity

**Figure 2:**
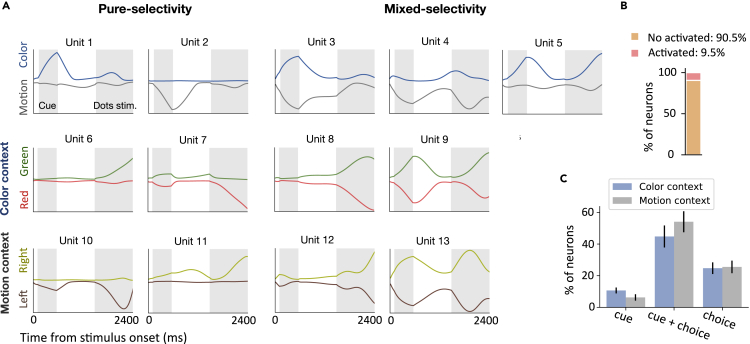
Single unitresponses (A) Single unit responses under different task conditions, as indicated by different colors. Two shaded areas represent the period of contextual cue stimulus presentation and sensory stimulus presentation, respectively. *Left panel*: Responses of units showed pure selectivity to the task-related variable. Unit 1 preferred the color cue, unit 2 preferred the motion cue, unit 6 preferred green, unit 7 preferred red, unit 10 preferred the leftward direction, and unit 11 preferred the rightward direction. *Right panel*: Different task-related variables were mixed. Unit 3 showed mixed selectivity for the color cue and motion cue. Units 4 and unit 5 showed mixed selectivity for both the context cue and sensory stimulus. Unit 8 showed mixed selectivity for two task variables (green and red) for the color context. Unit 12 showed mixed selectivity for two task variables (left and right) for the motion context. Unit 9 showed mixed selectivity for three task features, such as the color context cue, green, and red sensory stimulus. Similarly, unit 13 showed mixed selectivity for the motion context cue, leftward direction, and rightward direction. (B) The percentage of units in the trained RNN that were activated to perform a delay context-dependent decision-making task, averaging over all 16 trial conditions and 20 trained RNN models. (C) Among all the activated units, the percentage of units that was selective to the context cue, sensory input, and their combinations in color and motion context, respectively. Error bar indicates SEM over 20 different training configurations.

Mixed selectivity of PFC neurons is important for implementing complex cognitive functions, manifesting itself as an ‘adaptive coding’ strategy (Duncan, 2001). We found that many units of the trained RNN exhibited mixed selectivity for task-related variables (Figure 2A). A unit was said to be selective to a task-related variable if it responded differently to the values of the parameters characterizing that variable. We classified the units based on their responses to different task parameters, and found four distinct types of mixed-selective units from the trained RNN. (1) Some units (about 11.6%) exhibited mixed selectivity to task rules (both color context cue and motion context cue), such as unit 3. (2) Some units (about 11.3%) exhibited mixed selectivity to both color sensory stimuli (red and green), such as unit 8. (3) Some units (about 21.8%) exhibited mixed selectivity to both directions of coherent motion, such as unit 12. (4) Some units (about 21.6%) exhibited selectivity to both task rules and sensory stimuli. For example, unit 5 and unit 9 responded to both the color cue stimuli and color sensory stimuli. Unit 4 and unit 13 responded to both the motion cue stimuli and motion sensory stimuli.

Although single-unit activity could be tuned to mixtures of multiple task-related variables, some other units were modulated primarily by only one of the task variables (what we will call ‘pure-selectivity’ units). For example, unit 1 was primarily selective to the color context cue, and unit 2 was selective to the motor cue. Some units only responded to sensory stimuli, such as unit 6, unit 7, unit 10, and unit 11 (Figure 2A). In the trained RNN, only a small subset of units showed activations to task variables (Figure 2B). An RNN unit was considered active if there was a time point where the instantaneous firing rate was greater than 5 Hz. Among those activated units, the number of units encoding the choice was much more than that of units encoding the context cue (Figure 2C). One possible explanation is that it was more difficult to integrate noisy sensory information than to distinguish the context information, so more units were recruited to process sensory information.

We have mainly introduced four types of mixed selectivity units, with varying degrees of selective responses to different task-related variables. However, the diversity of mixed selectivity responses often resulted in response properties that were not easily interpretable. For example, in the color context, unit 9 simultaneously responded to the color cue stimulus and green signal, which also responded to the red signal. Specifically, units with such mixed selectivity behaved the same way in the same context, causing a difficulty of interpretation. This suggests that the activity of individual mixed selectivity units could not fully disambiguate the information; only when pooling information from multiple units, the ambiguity of information encoded by mixed selective units can be eliminated, supporting the necessity of population coding by ensembles of neurons (Rafael, 2015).

### Population Response

We further studied the neural representation at the population level. The dynamics of population activity can be characterized through the high-dimensional state space $x\left(t\right)\in R^{N}$. The time-varying population activity can be visualized as a trajectory within the lower-dimensional subspace, and the distance between the points in the subspace reflects the population response difference.

#### Cue processing

**Figure 3:**
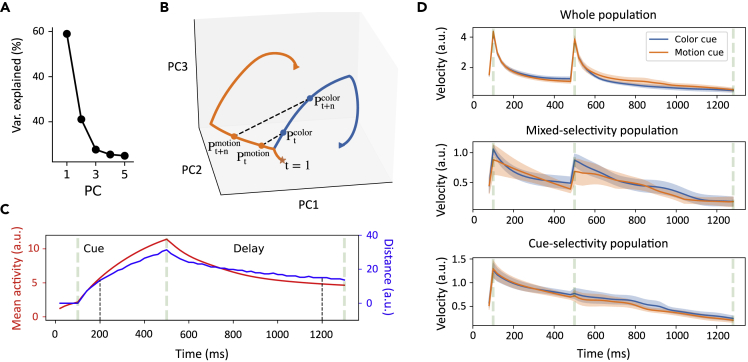
Neural population dynamics (A) Ratio of explained variance of the first five PCs of neural subspace during the cue stimulus and delay epochs. (B) Two different neural trajectories in a three-dimensional subspace during the cue stimulus and delay epochs. Orange and blue curves correspond to the motion and color contexts, respectively. The three-dimensional distance between two context-specific states at time *t* characterized the similarity of their population responses: $dist\left({\text{P}}_{\text{t}}^{\text{color}},{\text{P}}_{\text{t}}^{\text{motion}}\right)$. In a context-specific state trajectory, we calculated the velocity, which quantify the change in trajectory position as a function of time. It is calculated by equation: $dist\left({\text{P}}_{\text{t}}^{\text{color}},{\text{P}}_{\text{t}+\text{n}}^{\text{color}}\right)/n$, in which $dist\left({\text{P}}_{\text{t}}^{\text{color}},{\text{P}}_{\text{t}+\text{n}}^{\text{color}}\right)$ represents the distance between states at time *t* and t+n. (C) Distance between two context-specific trajectories in the cue stimulus and delay epochs as a function of time (blue curve). For comparison, the overall mean network activity (or network energy) is shown in a red curve, which is defined as the average firing rate of all activated units. (D) The top panel plots the velocity of the temporal evolution of overall population state ($\left|\dot{x}\right|$) under color context (blue) and motion context (orange). Shaded area denotes SEM over 20 training configurations. The middle and bottom panels show the state velocity evolution of mixed-selectivity population and cue-selectivity population, respectively.

To examine cue processing, we reported the dynamics of population activity throughout the cue stimulation and delay epochs. We performed principal component analysis (PCA) to identify a three-dimensional neural subspace, which was spanned by the first three principal components (PCs) that accounted for about 92% of neural activity variance (Figure 3A). During the cue presentation epoch, the network started at the same subspace and then evolved along different trajectories based on the contextual cues (Figure 3B). Further, the distance between two state trajectories increased (Figure 3C, blue curve), indicating that these two trajectories were continuously divergent during the cue stimulus epoch. Moreover, we defined the “energy” of the population activation state using the average activity of the total number of activated units (Figure 3C, gray curve).

We found that the distance curves between two cue-specific trajectories was temporally aligned with the increase of energy level during the cue stimulus and delay epochs. During the delay epoch, the network settled in a low-energy state. Moreover, the distance between two trajectories reached a peak value at the end of the cue stimulus presentation, then decreased during the delay epoch until reaching a plateau. However, the plateau value was greater than zero (a similar level as at time t=200 ms), suggesting that the difference in cue-specific trajectories still existed to distinguish the context conditions.

We computed the “velocity” of population activity, which is defined as the change in trajectory position as the function of time (Stokes et al., 2013). In the figure illustration (Figure 3B), it can be calculated by equation $dist\left({\text{P}}^{\text{t}},{\text{P}}^{\text{t}+\text{n}}\right)/n$, in which $dist\left({\text{P}}^{\text{t}},{\text{P}}^{\text{t}+\text{n}}\right)$ represents the distance between states at time *t* and t+n for a given context. Before the cue stimulus appeared, the overall population activity had a rapid acceleration to reach a peak (Figure 3D, top panel), and then the velocity of population activity gradually decayed to the pre-stimulus baseline level, suggesting that cue-specific trajectories were separated at a stable velocity in the late stage of cue-stimulus. During the delay epoch, the velocity of population activity jumped to a large value again and then dropped rapidly. The phenomenon was primarily contributed by mixed-selective units. Specifically, we plotted the respective velocities of population activity with pure-selectivity and mixed selectivity for comparison (Figure 3D). We observed that the velocity of the population that was only sensitive to the context cue decreased continuously throughout the cue stimulus and delay epochs. In contrast, for the mixed selectivity population, there was a jump point in the velocity curve at the beginning of the delay epoch. Therefore, the velocity is sensitive to change of epoch-wise population activity, and provides an informative measure of the population dynamics.

Since there is no sensory input during the delay epoch, the cue information must be stably stored as WM to guide the subsequent decision-making. A related question is how the model performance will be affected subject to weight perturbation. Motivated by the local optogenetic manipulation in animal experiments (Gray et al., 2017), we conducted weight perturbation by up- or down-scaling the recurrent connection weights with different scale values. Figure S2 illustrates a global perturbation, in which the weights of trained RNN are scaled by the same constant across all task epochs. In contrast, Figure S3 illustrates a local perturbation, in which the weights were only scaled locally in time during the delay epoch. We found that a small degree of perturbation did not affect the RNN’s ability to perform tasks. Additionally, the RNN performance was more robust with local perturbation than with global perturbation.

#### Sequential activity

**Figure 4:**
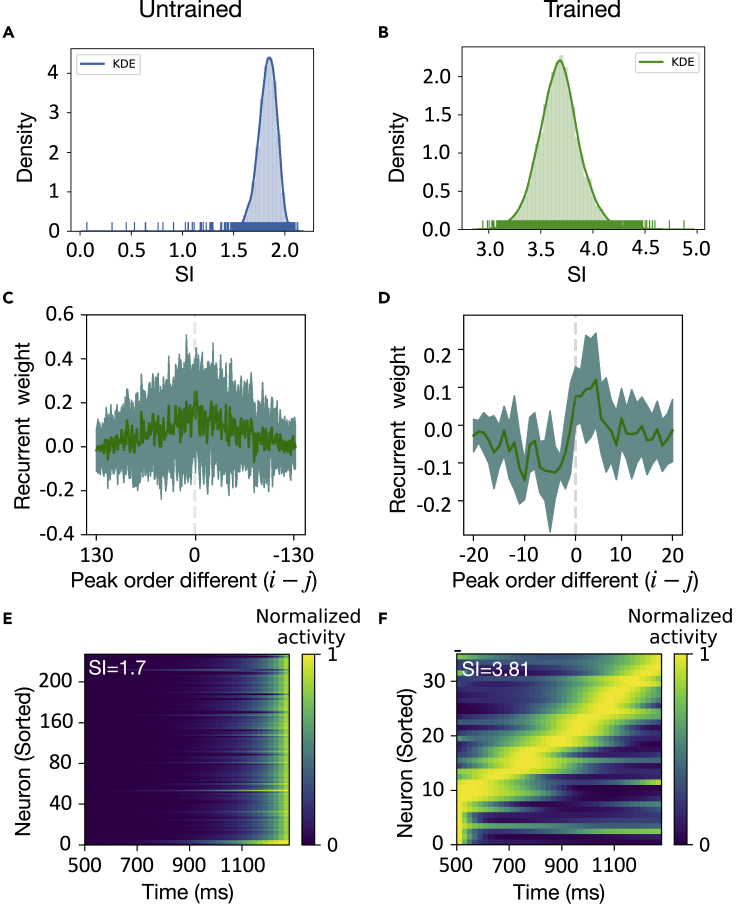
Sequential neural representation (A and B) The Monte Carlo distribution of sequentiality index (SI), which was computed by 10,000 samples from repeated random sampling. SI was computed from the untrained (A) and trained (B) networks. (C and D) Units were sorted according to their peak time in untrained (C) and trained (D) networks. The recurrent weights $\left(W_{ij}\right)$ were plotted as a function of the peak order difference between pre- and post-synaptic units during the cue-delay epoch. A positive (i−j) value represents a connection from a pre-synaptic to a post-synaptic unit. A negative (i−j) value represents a connection from a post-to a pre-synaptic unit. Both mean (solid lines) and standard deviation (shaded regions) statistics were computed from multiple trained networks. (E and F) Heat maps of normalized unit activity normalized by the peak response (per row) and sorted by the peak time. The activity did not appear ordered in an untrained network (E), whereas sequential activity emerged in the trained network (F).

Neural sequences are emergent properties of RNNs in many cognitive tasks, which has been thought to be a common feature of population activity during a wide range of behaviors (Fiete et al., 2010; Rajan et al., 2016; Orhan and Ma, 2019; Rajakumar et al., 2021). We found that our trained RNN generated emergent sequential activity during the delay epoch (Figure 4F). To quantify the sequentiality of neural activity, we calculated the sequentiality index (SI). Briefly, the SI is defined as the sum of the entropy of the peak response time distribution of the recurrent neurons and the mean log ridge-to-background ratio of the neurons, where the ridge-to-background ratio for a given neuron is defined as the mean activity of the neuron inside a small window around its peak response time divided by its mean activity outside this window (Orhan and Ma, 2019). By virtue of random sampling and Monte Carlo statistics, we found that the trained network has a higher SI (p<0.017, two-sample Kolmogorov-Smirnov test) than the untrained network (Figures 4A and 4B), suggesting that stronger sequential activity emerged from a trained network (Figure 4F).

Next, we investigated the computational mechanism that produces neural sequential activity. Similar to Rajan et al. (2016), we ordered the peak firing time of recurrent units and computed the mean and standard deviations of the recurrent weights $W_{ij}^{rec}$. This mean statistic was plotted as a function of the order difference (i−j) between the *i*-th and *j*-th units (Figures 4C and 4D). Interestingly, the connection weight of trained RNN showed an asymmetric peak, that is, the connection in ‘forward’ direction (*i.e.*, from earlier-peaking to later-peaking units) was strengthened more than those in ‘backward’ direction (*i.e.*, from later-peaking to earlier-peaking units) (Figure 4D). Therefore, this asymmetrical peak weakened the connections between temporally distant units, while strengthening the connections between temporally close units. However, this asymmetric structure was absent in the untrained network (Figure 4C), resulting in the loss of sequential activation structure (Figures 4E and S4). Put together, the asymmetric weight profile in the trained RNN could prolong responses in later-peaking units, producing the emergent sequential activity.

#### Rotation dynamics

**Figure 5:**
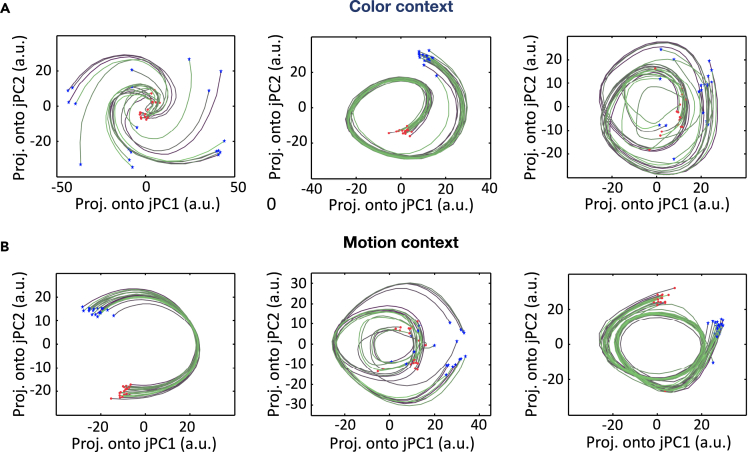
jPCA projectionsof the population response during the delay Epoch (A) Three examples of two-dimensional population rotational dynamics in the color context, which corresponded to different training configurations. Each trace represents one computer simulation trial (blue star: trial start point; red circles: trial endpoint). (B) Three examples of two-dimensional population rotational dynamics in the motion context.

The oscillatory nature of dynamical system is sometimes characterized by the so-called rotation dynamics, in which the sequential activity of neural populations can be approximated follow a rotating trajectory through their state space. The rational dynamics was initially reported in the motor cortex (Churchland et al., 2012). In recent year, researchers have uncovered rotational dynamics more broadly in other cortical areas, such as the auditory cortex (Libby and Buschman, 2021), and PFC (Aoi et al., 2020). The jPCA is a dimensionality reduction technique that finds an oscillatory structure in the data (Churchland et al., 2012). We performed jPCA on population responses in different contexts and visualized the two-dimensional projections of responses in the jPCA space (Figure 5). Interestingly, the trained RNN showed a strong rotation dynamic similar to those observed (Sussillo et al., 2015). Moreover, our result supported the findings of Lebedev et al., 2019, which showed that the rotation dynamics is essentially a “by-product” of sequential activation of population activity.

### Definition of task epoch-specific subspaces and axes

**Figure 6:**
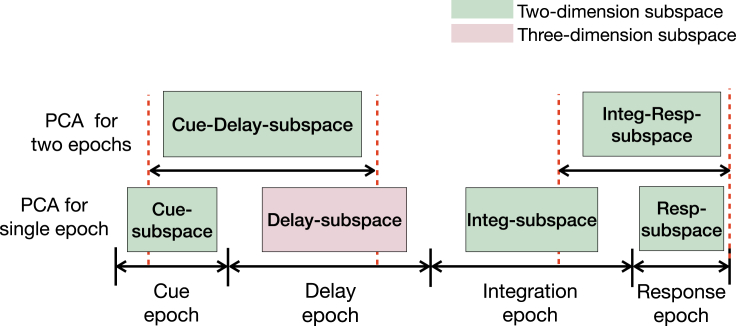
Illustration of distinct subspaces generated in different task Epochs Six subspaces are obtained by performing epoch-wise PCA on population response activities during six different periods, including four single task epochs (Cue epoch, Delay epoch, Integration epoch, and Response epoch) and two cross-epochs (Cue-Delay epoch and Integration-Response epoch).

To probe how neural population activity dynamically encoded task-related variables, we analyzed the population responses during six different task periods, including four single task epochs (cue stimulus epoch, delay epoch, integration of sensory stimulus epoch, and response epoch) and two cross-epoch periods (Figure 6). As expected, the correlation between population firing during these six periods varied (Figure S6).

We first performed epoch-wise PCA on the population response at single task epochs to generate the corresponding state subspaces (*i.e.*, Cue-subspace, Delay-subspace, Integ-subspace, and Resp-subspace). To characterize the evolution of trajectory, we further defined four task-related axes (Table 1): the axis of color context cue (C-cue-axis), the axis of motion context cue (M-cue-axis), the axis of color choice (C-choice-axis), the axis of motion choice (M-choice-axis). The definitions of these task epoch-specific subspaces and axes provide a geometric framework for population response analyses. Next, we projected these four task-related axes onto the corresponding state subspaces and examined the neural trajectory in a subspace-specific manner.

**Table 1:** The definition of geometry concepts in five neural subspaces. The matrix **X** varies according to different subspace.

Figure	Subspace(in two dimensions)	Geometric notion	Definition and interpretation
Figure 7A	Cue-subspace	C-cue-axis M-cue-axis	PCA on matrix X_*cue,color*_*,* the first PC is defined as the C-cue-axis. PCA on matrix X_*cue,motion*_, the first PC is defined as the M-cue-axis.
		cue-PC1 cue-PC2	PCA on population responses during the cue stimulus epoch. The first three PCs are: cue-PC1, cue-PC2, cue-PC3.
Figure 7C	Cue-Delay-subspace	cue-delay-PC1 cue-delay-PC2	PCA on population responses across two task epochs (Figure 6). The first three PCs are: cue-delay-PC1, cue-delay-PC2, cue-delay-PC3
Figure 8D	Integ-subspace	C-choice-axis M-choice-axis	PCA on matrix X_*integ,color*_, the first PC is defined as the C-choice-axis. PCA on matrix X_*integ,motion*_, the first PC is defined as the M-choice-axis
		integ-PC2 integ-PC3	PCA on population responses during the sensory stimulus epoch. The first three PCs are: integ-PC1, integ-PC2, integ-PC3.
Figure 9B	Resp-subspace	resp-PC2 resp-PC3	PCA on population responses during the response epoch (Figure 6). The first three PCs are: resp-PC1, resp-PC2, resp-PC3.
Figure 9D	Integ-Resp-subspace	integ-resp-PC2 integ-resp-PC3	PCA on population responses across two task epochs (Figure 6). The first three PCs are: integ-resp-PC1, integ-resp-PC2, integ-resp-PC3.

**Figure 7:**
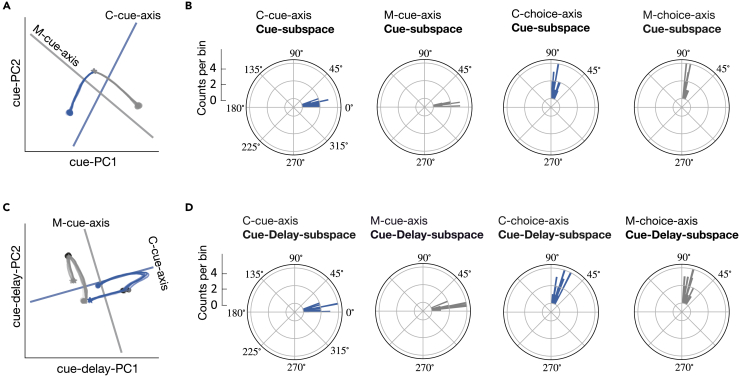
Visualization of neural trajectory in task subspaces (A) Neural trajectory in the Cue-subspace during the cue stimulus epoch. Blue and gray curves correspond to the color and motion contexts, respectively. Stars and circle indicate the start and endpoints of the cue stimulus epoch, respectively. Solid lines represent the projections of the C-cue-axis (blue) and M-cue-axis (gray) in this subspace. (B) Polar histograms of the angles between four task-related axes and Cue-subspace during the cue stimulus epoch over 20 training configurations. (C) Neural trajectory in the combined Cue-Delay-subspace. Stars denote the neural state at 100 ms after cue stimulus onset, circles denote the beginning points of the cue-delay epoch, and dark circles denote the state at 200 ms before the end of delay. (D) Polar histograms of the angles between four task-related axes and Cue-Delay-subspace.

First, we performed PCA on the population response during the cue stimulus epoch, and the first two principal components (cue-PCs) explained 93.1% of data variance (Figure S6A). This indicates that trajectories in the two-dimensional subspace (denoted as Cue-subspace spanned by cue-PC1 and cue-PC2) captured the majority of variance of population responses. We had a similar finding in the Cue-subspace as shown in Figure 3B: The network state started from the same location and then produced different trajectories according to different cue stimulus conditions (Figure 7A). For a given context, the neural state evolved along a stereotypical trajectory across all sensory stimuli.

Next, we calculated the angle between Cue-subspace and four predefined axes (*i.e.*, C-cue-axis, M-cue-axis, C-choice-axis, and M-choice-axis), respectively. Geometrically, if the angle between an axis and a plane is greater than $70^{\circ }$, they are considered nearly orthogonal; if it is less than $20^{\circ }$, they are considered nearly parallel or overlapping. We found that the angle between C-cue-axis and Cue-subspace, as well as the angle between M-cue-axis and Cue-subspace, were both less than $20^{\circ }$ (Figure 7B). This indicates that the space constructed by the C-cue-axis and M-cue-axis was overlapping with Cue-subspace. However, the angle between the C-choice-axis and Cue-subspace was around $70^{\circ }$-$90^{\circ }$, as well as the angle between the M-choice-axis and Cue-subspace was both around $70^{\circ }$-$90^{\circ }$. Therefore, both the C-choice-axis and M-choice-axis were nearly orthogonal with Cue-subspace. Based on these observations, we only projected C-cue-axis and M-cue-axis onto Cue-subspace (Figure 7A). We found that in the color context, all trajectories moved along the C-cue-axis but were insensitive to the M-cue-axis. Similarly, in the motion context, all trajectories moved along the M-cue-axis but were insensitive to the C-cue-axis. Furthermore, the angle between M-cue-axis and C-cue-axis mostly centered around $75^{\circ }$-$90^{\circ }$ (Figure S6B), suggesting that these two contextual cues were represented in two almost orthogonal subspaces.

### Cue information maintenance

We further explored the question: What is the relationship between neural trajectories across task epochs? The answer to this question can help us understand how the cue information generated in the cue stimulus epoch are preserved during the delay epoch. Specifically, we performed PCA during a 1000 ms time window starting at 100 ms after the cue stimulus presentation and ending at 200 ms before the end of delay epoch. We examined the state trajectory in the two-dimensional space (denoted as Cue-Delay-subspace) spanned by the first two principal components (cue-delay-PC1, cue-delay-PC2), which explained 75% variance (Figure S6D). Meanwhile, we projected four predefined axes onto Cue-Delay-subspace (Figure 7C), and then calculated the angle between the axes and Cue-Delay-subspace (Figure 7D).

Figure 7C describes the evolution of the population dynamics in relation to cue stimulus and delay epochs. To depict the transition of population dynamics more clearly, we further divided the 1000-ms time window into two intervals. The first 300-ms interval was within the cue stimulus period, starting from the 100 ms after the cue stimulus onset until the end of cue stimulus. During this interval, the separation between two context-specific trajectories (blue for the color context, gray for the motion context) diverged along their corresponding context cue axes, and reached a maximum at the end of this interval. In the follow-up 700-ms window within the delay epoch, the separation between two context-specific trajectories remained converged along their corresponding context cue axes. Therefore, the separation of context information generated in the cue stimulus epoch was preserved in the delay epoch, even if this separability weakened during the delay epoch. Moreover, for the given context, the projection of trajectories in the C-cue-axis at t=100 ms was almost the same as that at t=1000 ms. This correspondence was consistent with the correspondence in the energy of population activity (Figure 3C, gray curve).

### Cue-dependent processing of sensory stimulus

**Figure 8:**
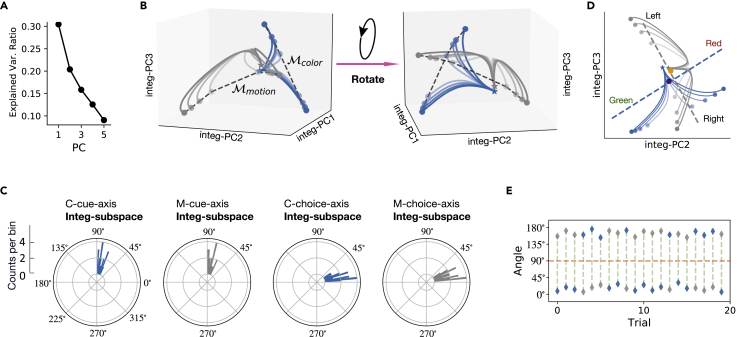
Population dynamics during the sensory stimulus Epoch (A) Ratio of explained variance of the first five PCs in Integ-subspace. (B) Neural trajectories in the three-dimensional subspace spanned by integ-PC1, integ-PC2, and integ-PC3. The blue and gray curve sets correspond to the neural trajectories in the color and motion contexts, respectively. Stars and circles indicate the start points and endpoints, respectively. The black dash curves connecting the endpoints mark manifold $M_{color}$ and $M_{motion}$, respectively. (C) Polar histograms of the angles between four task-related axes and Integ-subspace. (D) Neural trajectories in the two-dimensional subspace spanned by integ-PC2 and integ-PC3. The blue dashed line represents the projection of the C-cue-axis and the dark blue solid circle denotes the center point of the projection. The gray dashed line represents the projection of the M-cue-axis and the orange solid circle denotes the center point of the projection. (E) The angles between integ-PC1 and the C-cue-axis (blue) or M-cue-axis (gray) in 20 trials.

Furthermore, we studied how population activity responded appropriately to sensory stimulus according to the current task context. Similarly, we applied PCA to the neural activity during the sensory stimulus epoch. The first three principal components (integ-PCs) explained 81% cross-trial variance (Figure 8A), which was caused by the strength and direction of the color evidence, the strength and direction of the motion evidence, and context information (color or motion). As shown by the evolution of the population dynamics in the three-dimensional subspace (Figure 8B), each neural trajectory corresponded to a specific task condition (the blue and gray curve sets correspond to the neural trajectories in the color and motion contexts, respectively), indicating that the activity trajectories in this subspace captured the relationship between the task-related variables.

To identify the Integ-subspace, we projected the trajectories into three possible subspaces: integ-PC1 and integ-PC2, integ-PC1 and integ-PC3, and integ-PC2, and integ-PC3. Among these projections of trajectories and choice axes, we selected the subspace defined by integ-PC2 and integ-PC3 that was most geometrically meaningful in a sense that the C-choice-axis and M-choice-axis is parallel to chosen Integ-subspace. We further restricted our analysis to the two-dimensional Integ-subspace (spanned by integ-PC2 and integ-PC3). We computed the angle between four task-related axes and Integ-subspace (Figure 8C). The angle between the C-cue-axis and Integ-subspace, as well as the angle between the M-cue-axis and Integ-subspace, both centered around $70^{\circ }$. This means that both the C-cue-axis and M-cue-axis were orthogonal with Integ-subspace. Moreover, the C-choice-axis and M-choice-axis were nearly orthogonal (Figure S8A). Further, the angle between the C-choice-axis and Integ-subspace, as well as the angle between the M-choice-axis and Integ-subspace, were both less than $30^{\circ }$. This implies that the subspace spanned by the C-choice-axis and M-choice-axis was overlapping with Integ-subspace; and the projections of the C-choice-axis and M-choice-axis onto the Integ-subspace were still orthogonal (Figure 8D). Next, we investigated the mechanism of selection and integration through examining the population responses in Integ-subspace in both two- (Figure 8D) and three-dimensional subspaces (Figure 8B). Several observations are in order.

First, the integration of sensory stimuli corresponded to an evolution of the neural trajectory during the presentation of stimulus signal. All trajectories started from the same starting point in three-dimensional subspace (Figure 8B), which corresponded to the initial pattern of population responses during the delay epoch. When the stimulus started, the trajectories quickly moved away from their initial state. In Integ-subspace, there was a distinct gradual evolution of the neural trajectory along the C-choice-axis and M-choice-axis (Figure 8D). Specifically, in the color (motion) context trials, the neural trajectory moved along two opposite directions, which corresponded to the two different visual targets, namely, green (left) and red (right).

**Figure 9:**
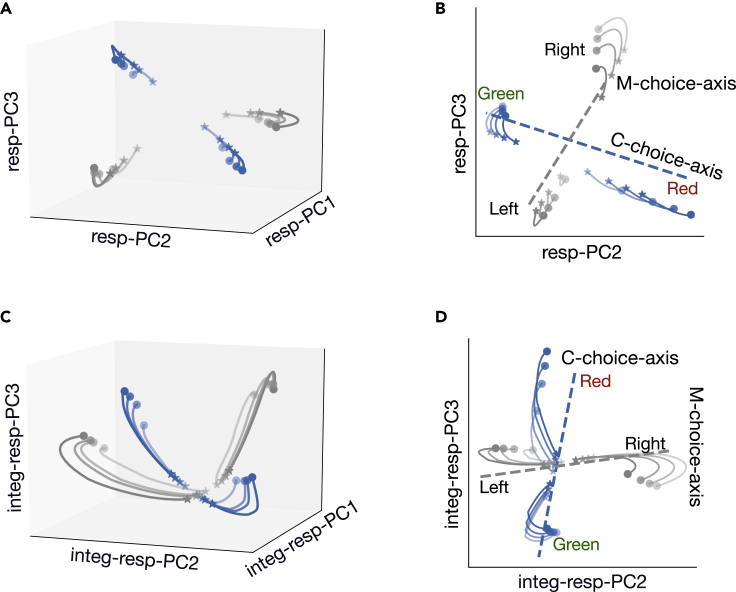
Visualization of neural trajectory in the response subspace (A) Neural trajectories in the three-dimensional subspace spanned by resp-PC1, resp-PC2, and resp-PC3 during the response epoch. (B) Same as panel A, except in two-dimensional subspace. (C) Neural trajectories in the three-dimensional subspace spanned by integ-resp-PC2, integ-resp-PC2, and integ-resp-PC3. Each trajectory started from 500 ms after the beginning of sensory stimulus until the action response. (D) Same as panel C, except in two-dimensional subspace.

Second, population responses varied according to different sensory inputs. Specifically, the neural trajectories corresponding to the color context were very different from those corresponding to the motion context, implying that sensory signals were separable at a context-dependent manner. In Figure 8D, the blue and gray curves represented neural trajectories in the color and motion contexts, respectively. In the color context, the patterns of population responses also varied with respect to different strengths and directions of the color stimulus. Therefore, neural trajectories captured multi-dimensional task-related variables, such as the context information, strength, and direction of sensory stimulus. Moreover, instead of following a straight line along the C-choice-axis or M-choice-axis, the neural trajectory formed an arc within the corresponding choice axis. The distance between the projection point of each arc onto the C-choice-axis and the color-center point (dark blue circle) reflected the strength of the corresponding color evidence while the position (two sides of the color-center point) of the projection point of each arc onto the color axis reflected the direction of the target (toward green or red). Once the stimulus signal disappeared, the network stopped to integrate the sensory evidence, yet the integrated evidence continued to be preserved along the C-choice-axis (Figures 9A and 9B). Similar discussions were also held in the motion context.

Third, population responses in the color and motion contexts occupied two different parts of subspace, which corresponded to two orthogonal-but-interesected subspaces. According to the above discussion, the neural trajectory in the color context was guided by the C-cue-axis and C-choice-axis. Therefore, the population activity during the color context occupied a subspace (*i.e.*, Color-subspace spanned by the C-cue-axis and C-choice-axis). In a similar fashion, population activity in the motion context occupied the Motion-subspace spanned by the M-cue-axis and M-choice-axis. We found that the projection of all neural trajectories onto an irrelevant choice axis was almost the same at each moment (Figure 8D). This indicates that the Color-subspace and Motion-subspace were mutually orthogonal: the sensory information in the color context could not be captured by the M-choice-axis, and the sensory information in the motor context could not be captured by the C-choice-axis. Moreover, we calculated the angle between the C-cue-axis and the integ-PC1, as well as the angle between the M-cue-axis and the integ-PC1. Noting that the range of this angle was $0^{\circ }$$∼180^{\circ }$, which helped us detect whether the projection directions of the two cue axes on integ-PC1 were opposite. We found at each trial, one angle was greater than $90^{\circ }$ and the other was less than $90^{\circ }$ (Figure 8E). That is, the projection directions of the C-cue-axis and M-cue-axis on integ-PC1 were always opposite, suggesting that Color-subspace and Motion-subspace were orthogonal but intersected.

### Task response

We applied PCA to the population activity during the response epoch, and found that the first three principal components (resp-PCs) explained 96% data variance (Figure S10A). The three-dimensional trajectories under different trial conditions occupied different positions in the Resp-subspace (Figure 9A). Similarly, we calculated the angle between the four task-related axes and the Resp-subspace (spanned by resp-PC2, resp-PC3). The result showed that: (1) The angle between C-cue-axis and Resp-subspace, as well as the angle between C-cue-axis and Resp-subspace, were both around $70^{\circ }$-$90^{\circ }$. (2) The angle between the C-choice-axis and Resp-subspace, as well as the angle between the M-choice-axis and Resp-subspace, both centered around $30^{\circ }$ (Figure S10B). Therefore, the near orthogonality between C-choice-axis and M-choice-axis could be maintained in Resp-subspace (Figure 9B). (3) The projection of the neural trajectory on the corresponding choice axis was almost the same at each moment, suggesting that there was no sensory evidence integration during the response epoch (Figure 9B).

To examine how the neural trajectory evolved from the sensory stimulus epoch to the response epoch, we reapplied PCA to the population activity during a combined-epoch 600-ms period: starting at 500 ms after the presentation of the sensory stimulus and ending at the moment of decision. The first three PCs (integ-resp-PCs) explain 92% cross-trial variance (Figure S11B). The neural trajectory in the two-dimensional subspace (denoted as Integ-Resp-subspace, Figure 9D) had similar characteristics to that during the sensory stimulus period: First, the angle between the C-choice-axis and Integ-Resp-subspace, as well as the angle between the M-choice-axis and Integ-Resp-subspace, both centered around $30^{\circ }$ (Figure S11A). Second, the projection directions of the C-cue-axis and M-cue-axis on integ-resp-PC1 were always opposite (Figure S11C), thus the integ-resp-PC1 fully captured the context cue information. Third, the projection of all neural trajectories on an irrelevant choice axis was the same at each moment (Figure 9D). Therefore, the population activity during this extended period also occupied two orthogonal-but-intersected subspaces.

Finally, we investigated the impact of weight perturbation on low-dimensional neural trajectories. Specifically, we perturbed the recurrent connection weights and examined the neural activity trajectory in the corresponding three-dimensional subspace. The range of local and global perturbation was restricted, and we examined the neural trajectories during the delay epoch and sensory stimulus epoch, respectively. Under a small weight perturbation, the relationship between task-related variables, such as the strength and direction of the stimulus, remained relatively robust (Figures S7 and S9). When the level of perturbation increased, the task-related information became lost gradually.

### A geometric interpretation of context-dependent integration

Based upon our geometric framework and subspace analyses, we propose a geometric interpretation for context-dependent computation during WM and decision making. To further illustrate the geometric notion, we defined dynamic attractors (such as fixed points) in the high-dimensional RNN dynamics (Sussillo and Barak, 2013).

**Figure 10:**
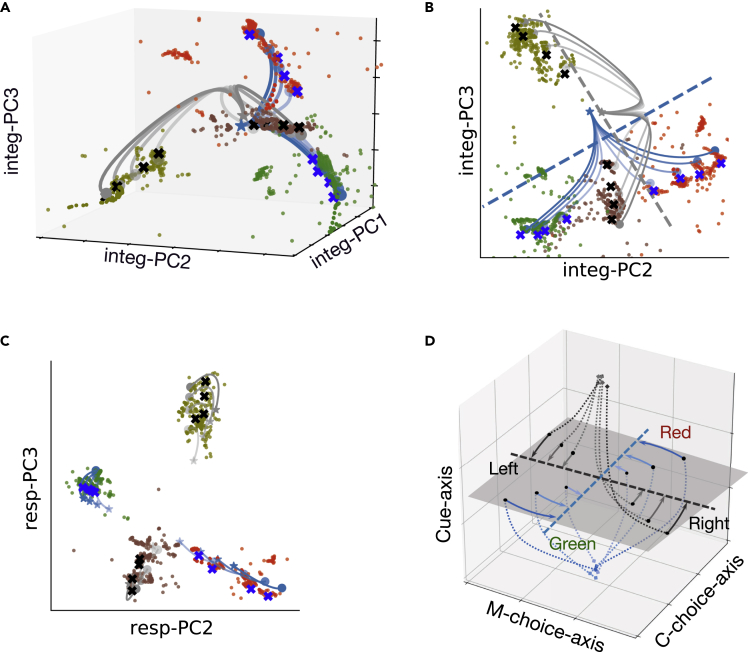
Visualization of dynamic attractors in neural subspace (A) Neural trajectories in three-dimensional Integ-subspace for different sensory stimuli eventually converged to different attractors. Blue and black crosses correspond to the color and motion contexts, respectively. Dots denote slow points and different colors correspond to different trial stimuli (red dots as red stimuli, green dots as green stimuli, olive dots as leftward direction stimuli, and brown dots as rightward direction stimuli). (B) The projection of slow points and fixed points onto Integ-subspace. (C) The projection of slow points and fixed points onto the Resp-subspace. (D) A schematic of neural trajectory through a three-dimensional subspace during the transition from evidence of sensory integration to choice making. Three axes include two choice axes and one context cue axis. Dotted lines represent neural trajectories during the sensory stimulus epoch, and solid lines reflect neural trajectories during a 75-ms window following the saccade onset.

Specifically, we used numerical optimization to minimize $q\left(x\right)=\frac{1}{2}{\left||\dot{x}|\right|}^{2}$ to identify the fixed points and slow points. Each dot in Figure 10A represents a slow point with q(x)<0.01, and each cross represents a fixed point with q(x)<0.0001. These fixed points were further arranged along a line for a specific context (blue crosses: color context; black crosses: motion context), forming a line attractor (Figures 10A and 10B).

We have showed the end of neural trajectories during the sensory integration epoch were aligned with manifold M (Figure 8B); and the projection of manifold M in the Integ-subspace was parallel to the corresponding choice axis (Figure 8D), suggesting that the color and motion information could be captured by manifold $M_{color}$ and manifold $M_{motion}$, respectively. Interestingly, the line attractors were aligned near the manifold, suggesting that the integration of sensory evidence could be explained by the arrangement of fixed points: During the sensory integration epoch, the population dynamics drove context-specific trajectories to back to their attractors associated with the correct choice. That is, the population activity was attracted toward the corresponding manifold M of slow dynamics at the end of the sensory stimuli (Figure 8B). By projecting these slow points and fixed points onto Resp-subspace, the population trajectories evolved around the corresponding fixed points (Figure 10C). Additionally, the sensory information integrated during the sensory stimuli epoch was maintained during the response epoch.

Moreover, our analysis within the proposed geometric framework has shown that Color-subspace and Motion-subspace were orthogonal-but-intersected. Therefore, the color contextual cue triggered a dynamical process so that the relevant color evidence was integrated while ignoring the irrelevant motion evidence from the same trials because of their mutual orthogonality in the subspace. The opposite pattern was also evident in the motion context.

Overall, we found clear geometrical structures in the task epoch-specific subspaces, which can provide (1) a new perspective to reexamine experimental data; (2) a quantitative framework to test computational hypotheses or mechanisms of context-dependent computation (3) an abstraction of theory (e.g., dynamic line attractor); for WM or decision making that supports experimental findings.

## Discussion

Context-dependent computation is a key hallmark for achieving cognitive flexibility. However, the computational principles underlying context-dependent WM or decision-making remains incompletely understood. We trained an RNN to perform a delayed context-dependent decision-making task, and proposed a geometric framework that helps uncover population dynamics of the trained RNN. Importantly, the trained RNN produced some emergent neurophysiological features at both single unit and population levels. The PCA and weight perturbation analysis further revealed neural representations of context-specific dynamic population coding and information integration. In low-dimensional neural subspaces, the RNN encoded the context information through the separation of neural trajectories and maintained the context information during the delay epoch. Finally, sensory integration during the decision-making period can be viewed by an evolving neural trajectory that occupies in two orthogonal-but-intersected subspaces.

Artificial RNNs are generally considered as black-box models and the underlying dynamical mechanisms are poorly understood. To unravel the black box, Sussillo and Barak (2013) proposed a dynamical systems framework to uncover the computational mechanisms of RNNs. In a similar manner, we used reverse-engineering and subspace analyses to uncover the mechanisms of population coding in the trained RNN. At the single unit level, many units exhibited mixed selectivity to different task-related variables. At the population level, sequential activation (‘neural sequences’) and rotational dynamics emerged from the trained RNN during the delay period.

In experimental data, neural sequential activity often emerged in temporally structured behaviors, which has been observed in the many brain regions, such as the mouse prefrontal or parietal cortices (Harvey et al., 2012; Schmitt et al., 2017). Moreover, several lines of RNN modeling work have been reported and our results of neural sequences and SI were also consistent with previous modeling (Orhan and Ma, 2019; Bi and Zhou, 2020). Notably, the experimental results reported by Mante et al. (2013) did not show sequential activity. One possible reason may be due to the fact that there was no temporal relationship between the contextual cue and the subsequent signal stimulus. Dimensionality reduction-based subspace analyses have proven useful to uncover temporal dynamics of RNNs (Kobak et al., 2016; Kao, 2019), and intuitive geometric notions can further reveal the orthgonality of task variables in the neural subspace (Bi and Zhou, 2020).

Our analyses demonstrated that the context cue triggered the separation of neural trajectories in a low-dimensional subspace, and the dynamic system was in a high-activity state. During the delay epoch, the population activity decayed to a stable low-activity state while maintaining the context separation. During the stimulus signal presentation, we found two main features of population responses: (1) The population responses exhibited different patterns to varying sensory inputs. (2) The population response in the color and motion contexts occupied two orthogonal-but-intersected subspaces. These features of population responses can be summarized schematically in Figure 10D, which provide fundamental constraints on the mechanisms of context-dependent computation in flexible cognitive tasks.

### RNNs for understanding computational mechanisms of brain functions

Due to its powerful computational capabilities, RNNs exhibit complex dynamics similar to experimental findings despite their oversimplification and abstraction of biological brain operation (nonlinear, feedback, and distributed computation) (Wolfgang et al., 2002; Sussillo and Abbott, 2009; Buonomano and Maass, 2009; Mante et al., 2013). In an analogue to animal behavioral training, RNNs can perform a wide range of cognitive tasks with supervised learning and labeled examples, including WM (Barak et al, 2010, 2013; Rajan et al., 2016), motor control (Laje and Buonomano, 2013; Hennequin et al., 2014), and decision-making (Song et al., 2016; Sussillo et al., 2015; Thomas, 2017; Yang et al., 2019; Aoi et al., 2020). The activity and network connectivity of the trained RNN can be accessed with specific perturbation strategies to help reveal underlying computational mechanisms. For example, the trained RNN can discover features of structural and functional connectivity that support robust transient activities bin a match-to-category task (Chaisangmongkon et al., 2017). Orhan and Ma (2019) identified the circuit-related and task-related factor that generates the sequential or persistent activity by training the RNN to perform various WM tasks. Goudar and Buonomano (2018) demonstrated that time-varying sensory and motor patterns can be stored as neural trajectories within the RNN, helping us understand the time-warping codes in the brain. RNNs have also been incorporated with more biological features, such as Dale’s principle, which serve as a valuable platform for generating or testing new hypotheses (Song et al., 2016; Xue et al., 2021; Rajakumar et al., 2021).

In many brain regions, information is encoded by the activity patterns of the neuronal population. A dynamical system view of population activity has become increasingly prevalent in neuroscience (Churchland et al., 2012; Sauerbrei et al., 2020; Shreya et al., 2020). With the help of dimensionality reduction, the population activity over time corresponds to neural trajectories in a low-dimensional subspace (Churchland et al., 2012; Kobak et al., 2016). Although we have focused our RNN modeling on a cognitive task, our geometric framework and subspace analysis can be applied to investigate other brain areas or brain functions, such as the premotor cortex or primary motor cortex in various motor tasks (Elsayed et al., 2016; Kao, 2019; Sussillo et al., 2015) and the striatum (Zhou et al., 2020).

### Relation to existing working memory theories

In context-dependent computational tasks, in order to decide which type of subsequent sensory inputs to be integrated, the information about the context rule needs to be preserved as WM across different task epochs. In the literature, various theories and computational models for WM have been developed. The classic models suggest that the PFC support WM via the stable, persistent activity of stimulus-specific neurons (Wang, 2001; Miller and Cohen, 2001; Miller, 2000), which bridges the gap between the memory representation of the context cue and sensory stimulus epochs. However, some experimental observations show that neural population coding during WM is dynamic (Barak et al., 2010; Spaak et al., 2017). Classical and new theoretical models in WM have been reviewed and tested in light of recent experimental findings (Lundqvist et al., 2016; Miller et al., 2018). Similar to other modeling efforts (Orhan and Ma, 2019; Xue et al., 2021), the single-unit response in our computer simulations showed strong temporal dynamics rather than persistent activity during the delay epoch. Therefore, our model supports the finding of experimental studies that information is often stored in dynamic population codes during WM. However, the WM is probably more complex than a simple theory that can explain everything, and neural representations (e.g., persistent vs. oscillatory dynamics) may highly depend on the specific task and recording area/technique. Moreover, unlike previous studies that suggested a dissociation between the stimulus-driven response and subsequent delay activity in the PFC (Barak et al., 2010; Meyers et al., 2008), our results showed the opposite phenomenon. In fact, the context information encoded by population activity during the cue stimulus epoch was maintained by low energy activity (Figure 3C) and sequential representation (Figure 4F) during the delay epoch.

In the trained RNN, population response is highly dynamic during both the cue stimulus and the delay epoch (Figure 3C). One possible explanation for the dynamic activity during the early part of the delay epoch is that it reflects the transformation from a transient context cue input into a stable WM representation, and this transformation is first contained in a high-energy dynamic trajectory in the state space, and then the system returns to a low-energy state. In the synaptic theory of WM (Mongillo et al., 2008), memories can be maintained as a pattern of synaptic weights, and neural activity changes synaptic efficacy, leaving a synaptic memory trace via short-term synaptic plasticity. This WM model suggests that the previous cue stimuli may be recovered from the network architecture, allowing for an energy-saving mode of short-term memory, rather than relying on the maintenance of high-energy persistent activity. Based on this synaptic theory of WM, Stokes (2015) have predicted that WM representations should be stationary and have low energy. Our RNN results are also consistent with this prediction.

### Limitations of the study

There are several limitations of our work. First, neurons in the mammalian cerebral cortex have diverse cell types and follow Dale’s principle—that is, they have excitatory and inhibitory effects on their postsynaptic neurons. However, such biological constraints were not considered in our current rate-based RNN model. Second, we trained the RNN using a standard gradient-based back-propagation algorithm, whereas synaptic plasticity in the brain is known to use Hebbian plasticity or spike-timing dependent plasticity (STDP). A combination of unsupervised and reinforcement learning algorithms to train RNNs would be more biologically plausible in modeling neuronal population dynamics. Third, finding line attractors in high-dimensional RNN dynamics relies on numerical methods and remains challenging. Development of efficient subspace methods for identifying dynamical attractors would be the subject of our future research direction.

## STAR★Methods

### Key resources table

**Table:** 

REAGENT or RESOURCE	SOURCE	IDENTIFIER
**Software and algorithms**		

Python	Open source	www.python.org
Sequentiality index	Orhan and Ma (2019)	https://github.com/eminorhan/recurrent-memory
Fixed point analysis	Sussillo and Barak (2013)	https://github.com/elipollock/EMPJ
Network structure	Yang et al. (2019)	https://github.com/gyyang/multitask
Dimensionality reduction and sequence visualization	Bi and Zhou (2020)	https://github.com/zedongbi/IntervalTiming
Deposited code	This study	https://github.com/Xhan-Zhang/contextInteg

### Resource availability

#### Lead contact

Further information and requests for data should be directed to and will be fulfilled by the Lead Contact, Zhe S. Chen (zhe.chen@nyulangone.org).

#### Materials availability

The study did not generate new reagents.

#### Data and code availability

All code has been deposited and is publicly available on GitHub (https://github.com/Xhan-Zhang/contextInteg)

### Method details

#### Network structure

We constructed an RNN network of N=256 fully interconnected neurons described by a standard firing-rate model. The continuous-time dynamics of the RNN are governed by the following equations

(Equation 1) $$\begin{array}{ll} \tau \dot{x} & =-x+W^{rec}r+W^{in}u+b+\sqrt{2\tau \sigma_{rec}^{2}}\xi ,\\ r & =f\left(x\right) \end{array}$$

where x, r, and u represent the synaptic current, firing rate, and network input, respectively; τ=20 ms is time constant, which mimics the synaptic dynamic on the basis of NMDA receptors; b is the background input; ξ are independent zero-mean Gaussian white noise scaled by $\sigma_{rec}=0.05$, which represent the intrinsic noise. The firing rate r is related to the corresponding current x by a Softplus transfer function f(x)=log(1+exp(x)), which maps every input currents to a positive firing rate. The output of the network is a linear mapping between the firing rate and a readout synaptic weight:

(Equation 2) $$z=W^{out}r+b^{out}$$

where $W^{in}$, $W^{rec}$, and $W^{out}$ are the input weight, recurrent weight, and output weight, respectively. We used Euler’s method to discretize the continuous-time equation and derive the discrete-time version

(Equation 3) $$\begin{array}{ll} \tau {\dot{x}}_{t} & =\left(1-\alpha \right)x_{t-1}+\alpha \left(W^{rec}r_{t-1}+W^{in}u_{t}+b+\sqrt{2\alpha^{-1}\sigma_{rec}^{2}}\xi \right)\\ r_{t} & =f\left(x_{t}\right) \end{array}$$

where ξ∼N(0,1) was drawn from a standard normal distribution; α=Δt/τ, and Δt is time step. In our study, we set Δt=20 ms. Similar to the previous computational models (Wang et al., 2018; Orhan and Ma, 2019), we used α=1. Furthermore, we assumed that the RNN received two types of noisy input: rule-specific input $u_{rule}$ and stimulus-specific input $u_{stim}$:

(Equation 4) $$\begin{array}{l} u=\left(u_{rule},u_{stim}\right)+u_{noise}\\ u_{noise}∼\sqrt{2\sigma_{in}^{2}}N\left(0,1\right) \end{array}$$

where $\sigma_{in}$ denotes the standard deviation of input noise. We set $\sigma_{rec}=0.05$, $\sigma_{in}=0.01$ in our computer simulations.

#### Task description

The task is schematized in Figure 1A, which is inspired and modified from a context-dependent task performed by macaque monkeys (Mante et al., 2013). The network received pulses from three input channels. The first channel consisted of the context cue stimulus, which contained two units encoding different context information. The other two channels consisted of two different sensory stimuli modalities. One channel represented the color sensory stimulus, which contained two units encoding the color stimulus strength $\gamma_{color,1}$, $\gamma_{color,2}$. The other channel represented the motion sensory stimulus, which encoded the motion stimulus strength $\gamma_{motion,1}$, $\gamma_{motion,2}$. The stimulus strengths were determined by the coherence for the color modality and motion modality ($c_{color}$, $c_{motion}$), and set as follows:

(Equation 5) $$\gamma_{color,1}=\bar{\gamma }+c,\;\gamma_{color,2}=\bar{\gamma }-c$$

A similar equation held for the motion modality. $\bar{\gamma }$ denotes the average strength of the two color stimuli, which was drawn from a uniform distribution $\bar{\gamma }∼U\left(0.8,1.2\right)$ (where U(a,b) represents a uniform distribution between *a* and *b*). Coherence *c* measured the strength difference of these two stimuli, which was uniformly distributed as

(Equation 6) c∼U(−0.08,−0.04,−0.02,−0.01,0.01,0.02,0.04,0.08)

The task consisted of distinct epochs. For each trial, a fixation epoch was present before the stimulus presentation. It was followed by the context cue stimulus epoch that lasted $T_{stim1}=400$ ms. After a delay epoch (with duration of $T_{delay}=800$ ms), the stimulus signal was presented in the second stimulus epoch with a duration of $T_{stim2}=800$ ms. Finally, the network responded in the Go epoch with an interval of $T_{resp}$.

#### RNN training

If the relevant evidence points towards choice 1, the output channel 1 (composed of two output units) was activated, otherwise the output channel 2 (composed of two output units) was activated. The cost function *L* is the mean squared error (MSE) between the network output (z) and target outputs ($\hat{z}$):

(Equation 7) $$L=\frac{1}{N_{out}}\overset{N_{out}}{\underset{i=1}{\sum }}{\left(z_{i}-{\hat{z}}_{i}\right)}^{2}$$

We optimized the weights $\left\{W^{in},W^{rec},W^{out}\right\}$ using the well-established Adam algorithm (Kingma and Ba, 2015), with default configuration of hyperparameters. The learning rate is 0.0005, and the exponential decay rate for the first and second moment estimates are 0.9 and 0.999, respectively. The off-diagonal connections of recurrent weight matrix $W^{rec}$ were initialized as independent Gaussian variables with mean 0 and standard deviations $0.3/\sqrt{N}$, and diagonal connections were initialized to 1. The initial input connection weights were uniformly drawn from −0.5 to 0.5. The output connection weights $W^{out}$ were initialized from an independent Gaussian random distribution with mean 0 and standard deviation $0.4/\sqrt{N}$.

#### Definition of task-related axes

We first grouped neural population activity into the matrix $X\in R^{N\times CT}$, where N=256 denotes the number of RNN units, C=1×8 denotes the number of the conditions (8 different sensory stimulus conditions in the given context), and *T* denotes the time step. Spike activity was binned by a 20-ms window. Different task epochs correspond to different X-matrices.

C-cue-axis and M-cue-axis. During the cue stimulus epoch, for the given color context, we obtained the matrix $X_{cue,color}\in R^{N\times CT}$, where N=256,T=400/20. We further performed PCA on the matrix $X_{cue,color}$. The first PC explained 91% of data variance (Figure S6C, green bar), so we defined it as the C-cue-axis. Similarly, for the given motion context, we performed PCA on the matrix $X_{cue,motion}\in R^{N\times CT}$ and the ratio of explained variance of the first five PCs is also shown in Figure S6C (grey bar). The first PC explained 92% of data variance. Therefore, we defined the first PC dimension as the M-cue-axis(Table 1).

C-choice-axis and M-choice-axis. The population activity matrices in the given context were $X_{integ,color}\in R^{N\times CT}$ and $X_{integ,motion}\in R^{N\times CT}$, where T=800/20. We performed PCA on $X_{integ,color}\in R^{N\times CT}$ and $X_{integ,motion}\in R^{N\times CT}$, respectively. The ratio of explained variance of the first five PCs in the specific context is shown in Figure S8B. In a similar fashion, we defined C-choice-axis and M-choice-axis for the color and motion contexts, respectively.

### Quantification and statistical analysis

#### Computation of sequentiality index (SI)

We computed the SI to quantify the sequential activation of the population response of excitatory neurons during the delay epoch. The SI is defined as the sum of the entropy of the peak response time distribution of the recurrent neurons and the mean log ridge-to-background ratio of the neurons, where the ridge-to-background ratio for a given neuron is defined as the mean activity of the neuron inside a small window around its peak response time divided by its mean activity outside this window (Orhan and Ma, 2019).

To compare the SI statistics of trained RNN with untrained RNN, we performed additional Monte Carlo control analyses. Briefly, given each randomly initialized weights, we simulate the activity of 256 neurons from the RNN using the same number of trials. We then averaged, sorted the population activity, and computed the SI statistic. We repeated the procedure 10,000 times and used that to compute the Monte Carlo p-value.

#### Finding rotation dynamics via jPCA

The jPCA method has been used to reveal rotational dynamics of neuronal population responses (Churchland et al., 2012). We assumed that the data were modeled as a linear time-invariant continuous dynamical system of the form: $\dot{x}=Mx$, where the linear transformation matrix M was constrained to be skew-symmetric (*i.e.*, $M^{⊤}=-M$). The jPCA algorithm projects high-dimensional data x(t) onto the eigenvectors of the M matrix, and these eigenvectors arise in complex conjugate pairs. Given a pair of eigenvectors $\left\{v_{k},{\bar{v}}_{k}\right\}$, the *k*-th jPCA projection plane axes are defined as $u_{k,1}=v_{k}+{\bar{v}}_{k}$ and $u_{k,2}=j\left(v_{k}-{\bar{v}}_{k}\right)$ (where $j=\sqrt{-1}$). The solution to the above continuous-time differential equation is given by $x\left(t\right)=e^{Mt}x\left(0\right)$, where the family $\left\{e^{Mt}\right\}$ is often referred to as the semi-group generated by the linear operator M. Since M is skew-symmetric, $e^{M}$ is orthogonal; therefore, it can describe the rotation of the initial condition x(0) over time. It is emphasized that the jPCA method is based on a linear approximation of the nonlinear dynamics described by the RNN, so that the potential rotation or oscillatory dynamics can be captured.

Applying eigenvalue decomposition to the real skew-symmetric matrix M, so that $M=U\Lambda U^{-1}$, where Λ is a diagonal matrix whose entries ${\left\{\lambda_{i}\right\}}_{i=1}^{N}$ are a set of (zero or purely imaginary) eigenvalues. Upon time discretization (assuming dt=1), we obtained the discrete analog of dynamic equation x(t+1)=(I+M)x(t). Alternatively, we directly solved a discrete dynamical system of the vector autoregressive (VAR) process form x(t+1)=Qx(t)over the space of orthogonal Q matrices. Mathematically, we have previously shown that this is equivalent to solving the following constrained optimization problem (Nemati et al., 2014):

(Equation 8) $$Q^{\text{∗}}=\text{arg}\underset{Q}{\text{min}}\left\|\right|A-Q{\left|\right\|}_{F}^{2},\text{subject to }QQ^{⊤}=I,$$

where $∥\cdot {∥}_{F}$ denotes the matrix Frobenius norm, and $A=\left(X_{t+1}X_{t}^{⊤}\right){\left(X_{t}X_{t}^{⊤}\right)}^{-1}$ represents the least square solution to the unconstrained problem x(t+1)=Ax(t). The solution to the above constrained optimization is given by the orthogonal matrix factor of Polar Decomposition of matrix A, namely A=QP (Higham, 1986).

#### Finding fixed points and line attractor

We focused on finding the fixed points and slow points of dynamical system to explore the mechanism by which the RNN performed the context-dependent task. The computational task was to find some points that satisfy $\dot{x}\left(t\right)=0$ for all *t*, that is, we needed to solve a first-order differential equation

(Equation 9) $$-x+W^{rec}r+W^{in}u+b+\sqrt{2\tau \sigma_{rec}^{2}}\xi =0$$

for a constant input u and with a transfer function r=f(x). However, the nonlinear function f(x) makes it difficult to find an analytical solution to the differential equation. Therefore, we identified the fixed points and slow points through numerical optimization. According to the algorithm described in Sussillo and Barak (2013), we solved the optimization problem as follows:

(Equation 10) $$\underset{x}{\text{min}}\;q\left(x\right)$$

where $q\left(x\right)=\frac{1}{2}{\left\|\dot{x}\right\|}^{2}$. To identify the local minimum, we defined the RNN state x as a slow point if q(x)<0.01 and x is a fixed point if q(x)<0.0001. These fixed points and slow points were calculated during the sensory stimulus epoch. The initial conditions for optimization were points in the neighborhood of x(t), which was the start state of RNN system trajectories. For each fixed point, we repeated the optimization procedure 300 times, and the initial conditions at each time were sampled from the neighborhood of x(t). Based on these 300 candidate fixed points, we chose stable fixed points as attractors, characterized by slow dynamics. We determined a fixed point being stable if neural states empirically converged to attractors. Here, we only considered stable fixed points (represented by cross symbols in Figure 10). For a given context, there were 8 different trial conditions, and the population activity on each trial condition was characterized by a state trajectory (Figure 8C). In total, we identified 16 attractors and each state trajectory eventually converged to its fixed-point attractor (Figure 10A). The initial conditions for slow point optimization were sampled from a normalized random Gaussian matrix. We repeated the optimization procedure 56 times for each trial condition and obtained 56×16=896 slow points.
