# Supplementary material for: A geometric framework for understanding dynamic information integration in context-dependent computation
Source: iScience. 2021 Jul 30;24(8):102919. doi: 10.1016/j.isci.2021.102919 (PMC8367843; doi:10.1016/j.isci.2021.102919)
Supplement: Document S1. Figures S1–S11 [file mmc1.pdf]

iScience, Volume *24*

Supplemental information

A geometric framework for understanding dynamic information integration in context-dependent computation

Xiaohan Zhang, Shenquan Liu, and Zhe Sage Chen

Supplemental Information

1 Titles of all supplemental figures and reference a main item

Figure S1.[Distributions of recurrent connection weights of trained RNN and the impact of the weight sparsity on the RNN performance] (**Related to Figure 1**)

Figure S2.[Performance of the trained RNN with respect to global perturbation] (**Related to Figure 1**)

Figure S3.[Performance of the trained RNN with respect to local perturbation] (**Related to Figure 3**)

Figure S4.[Normalized response of recurrent units in a set of examples from the untrained network] (**Related to Figure 4**)

Figure S5.[The correlation matrices of population activity in six task-specific periods.] (**Related to Figure 6**)

Figure S6.[Ratio of explained variance and the angle] (**Related to Figure 7**)

Figure S7.[The impact of weight perturbation on neural representation in the subspace] (**Related to Figure 7**)

Figure S8.[The angle and ratio of explained variance] (**Related to Figure 8**)

Figure S9.[The neural trajectories under recurrent weight perturbation with different SV values] (**Related to Figure 8**)

Figure S10.[Ratio of explained variance and the angle during the response epoch] (**Related to Figure 9**)

Figure S11.[Ratio of explained variance and the angle] (**Related to Figure 9**)

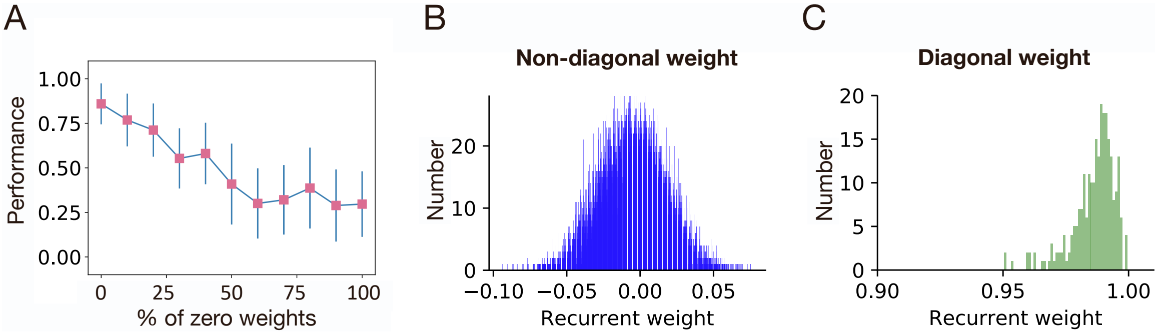

Figure S1: Distributions of recurrent connection weights of trained RNN and the impact of the degree of weight sparsity on the RNN performance.**(A)** The accuracy of RNN performance with respect to the percentage of zero weight entries. **(B)** Histogram of non-diagonal recurrent weight connections of the trained RNN. **(C)** Histogram of diagonal self-connection weights of the trained RNN.

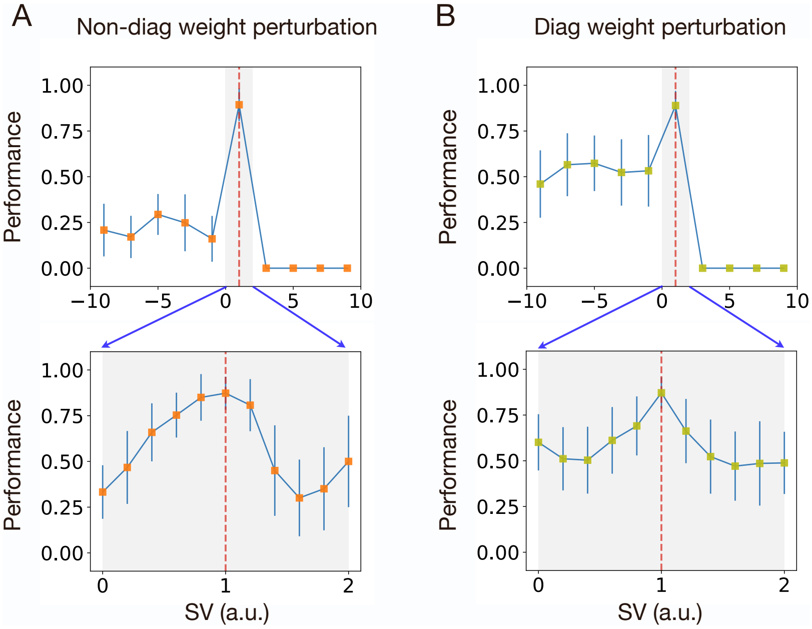

Figure S2: Performance of the trained RNN with respect to global perturbation, where weights were scaled by a constant across the complete task epochs. Perturbing the trained RNN by scaling the recurrent connection weights with different scale values (SVs). **(A)** The impact of non-diagonal weight perturbation on the performance. The red vertical dashed line corresponds to the non-perturbed RNN model ($SV = 1$). The error bars indicate SEM over 20 trained models. The zoom-in shade area is shown in the bottom panel. The trained RNN preserved a robust performance between $SV = 0$ and $SV = 1.2$. **(B)** Similar to panel A, but for diagonal weight perturbation. The trained RNN preserved a robust performance between $SV = 0.9$ and $SV = 1.1$.

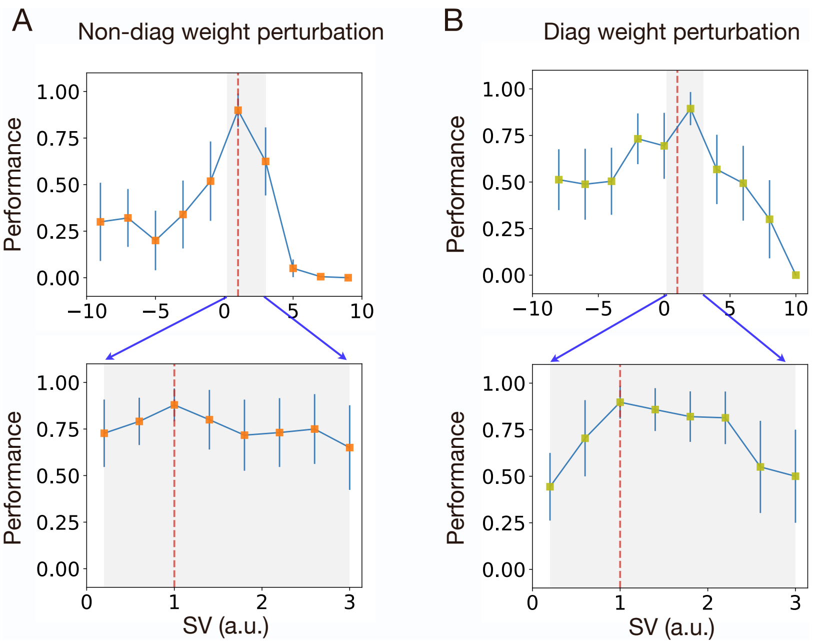

Figure S3: Performance of the trained RNN with respect to local perturbation, where weights were scaled by a constant locally during the task delay period only. Perturbing the trained RNN by scaling weights locally in time during the delay epoch. **(A)** The top panel plots the impact of non-diagonal connection weight perturbation on the performance. The network showed a robust performance between $SV = 0.5$ and $SV = 1.5$. **(B)** The impact of diagonal recurrent weight perturbation on the performance. The network showed a robust performance between $SV = 1.01$ and $SV = 2$.

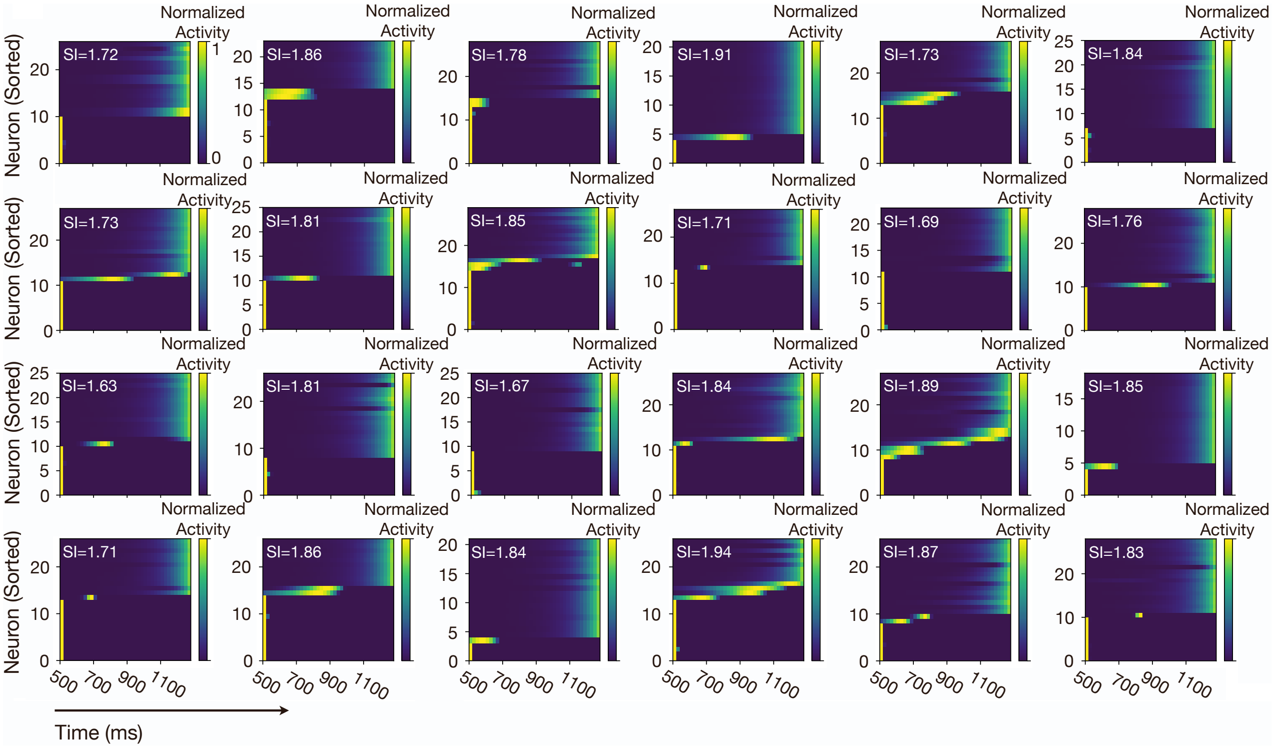

Figure S4: Selective 24 heat map examples of sorted population responses from 30 randomly selected RNN units. In each plot, 30 units were normalized by their maximal responses and sorted by the peak time.

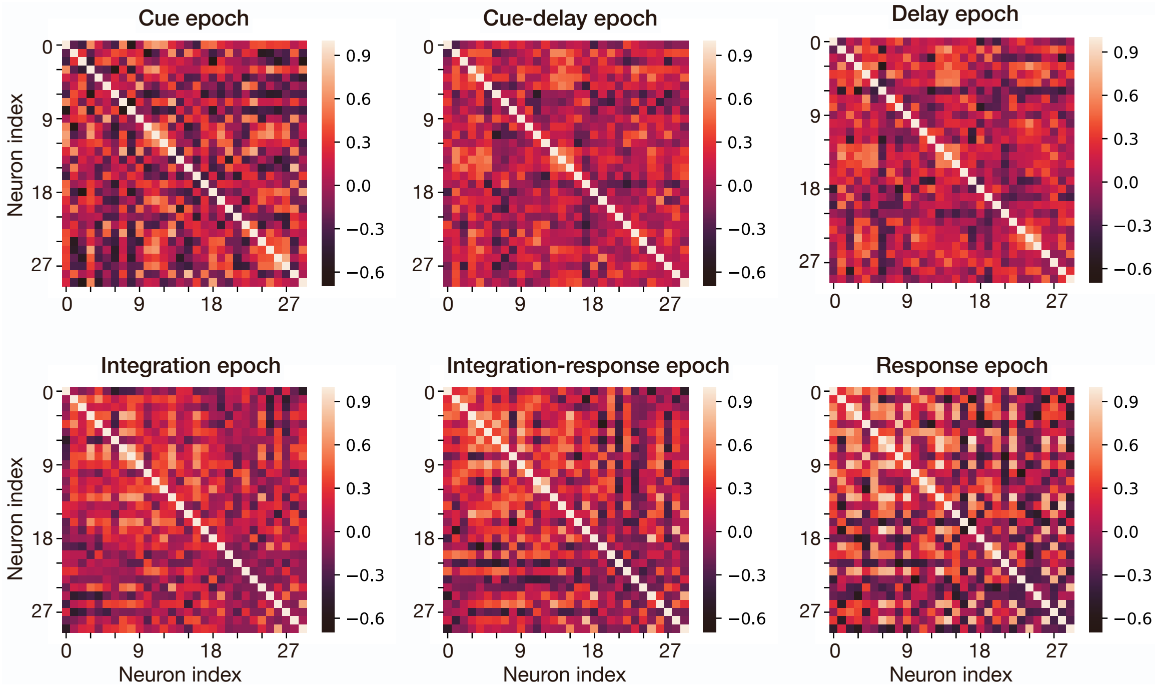

Figure S5: The correlation matrices of population activity in six task-specific periods. For clear visualization and comparison, we plotted 30 units with the higher mean activity across six epochs.

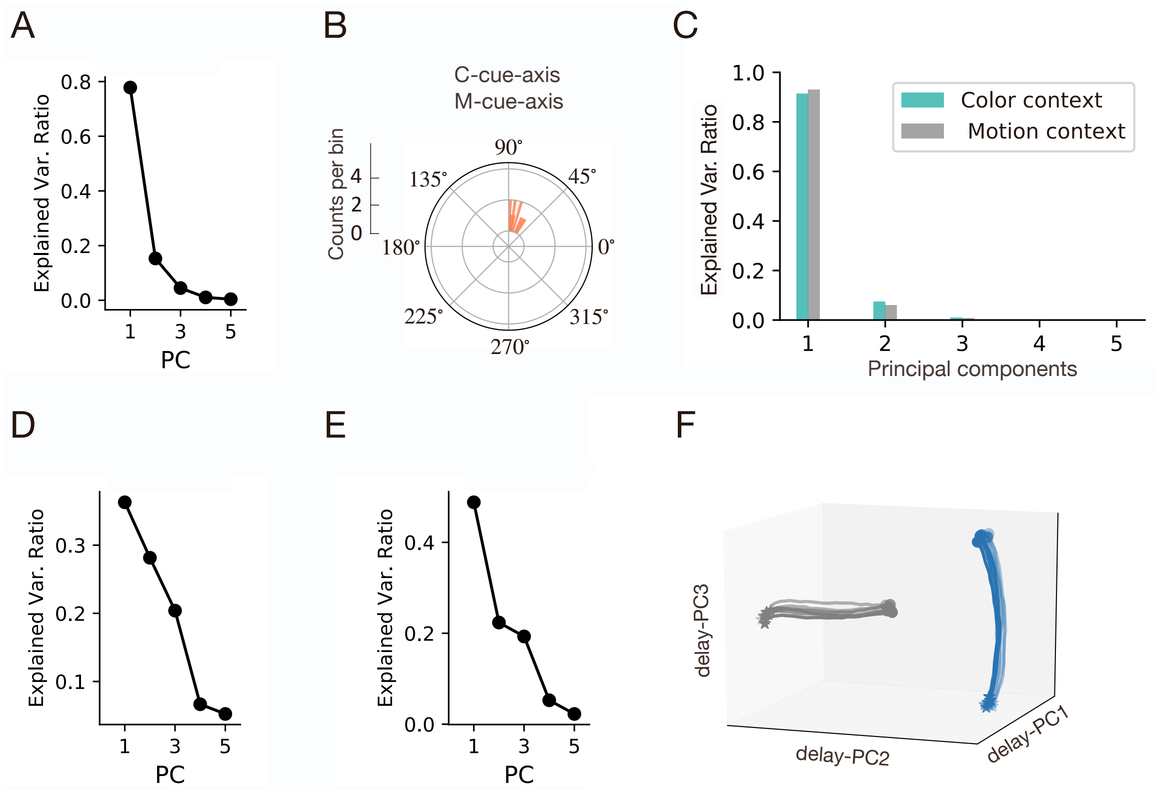

Figure S6: **(A)** Ratio of explained variance and the angle during the cue stimulus epoch. **(B)** Polar histogram of the angle between the `M-cue-axis` and `C-cue-axis`. **(C)** Ratio of explained variance of the first five PCs of subspace for the color and motion contexts, respectively. **(D)** Ratio of explained variance and the angle during a 1000-ms time window starting at 100 ms after the presentation of cue stimulus and ending at 200 ms before the end of delay. **(E)** Ratio of explained variance and the angle during the delay epoch. **(F)** Neural trajectories in the three-dimensional subspace spanned by delay-PC1, delay-PC2, delay-PC3.

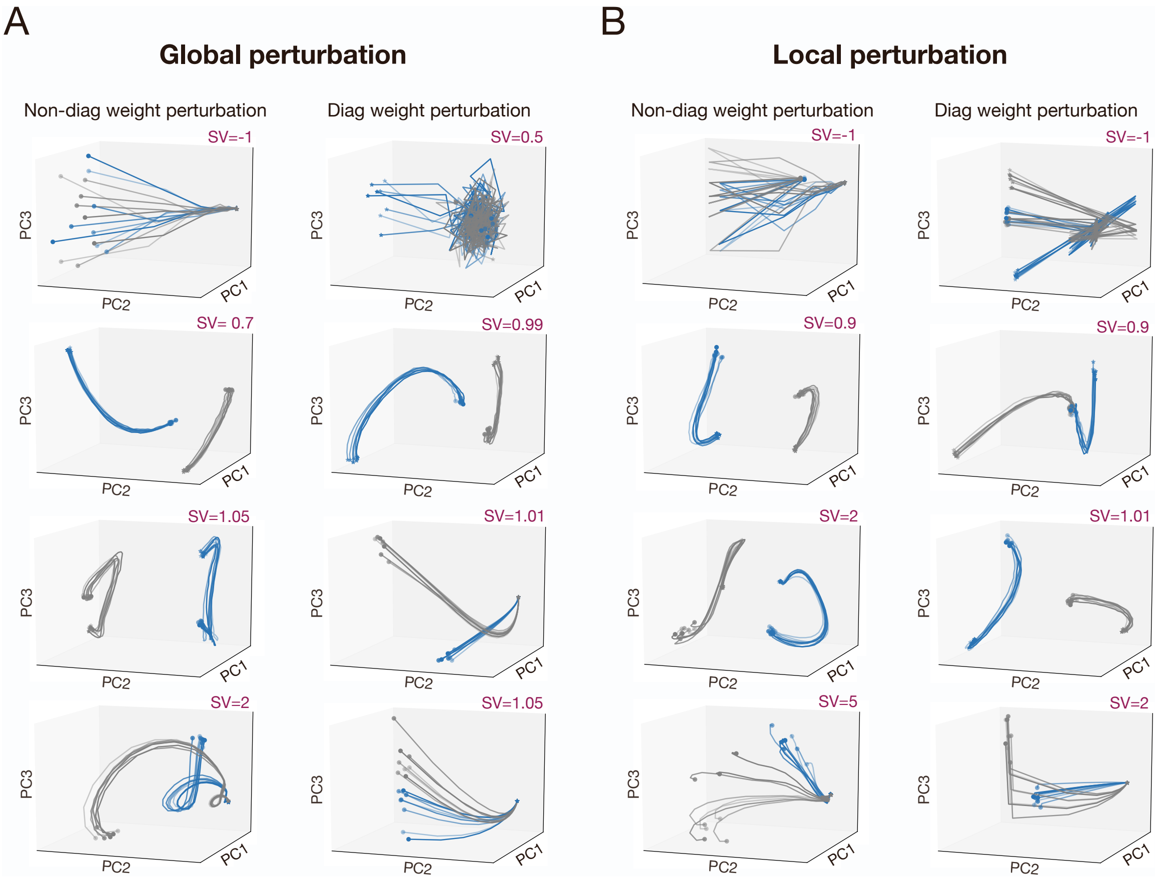

Figure S7: The impact of weight perturbation on neural representation in the subspace. The neural trajectory for four different SVs in a three-dimensional subspace, which is spanned by the first three PCs (delay-PC1, delay-PC2, delay-PC3). **(A)** Weight perturbation was operated during the complete task period, namely, global perturbation. The first/third-column panels indicate non-diagonal weight perturbation, and the second/fourth-column panels indicate diagonal weight. **(B)** Perturbation was operated only locally during the delay epoch.

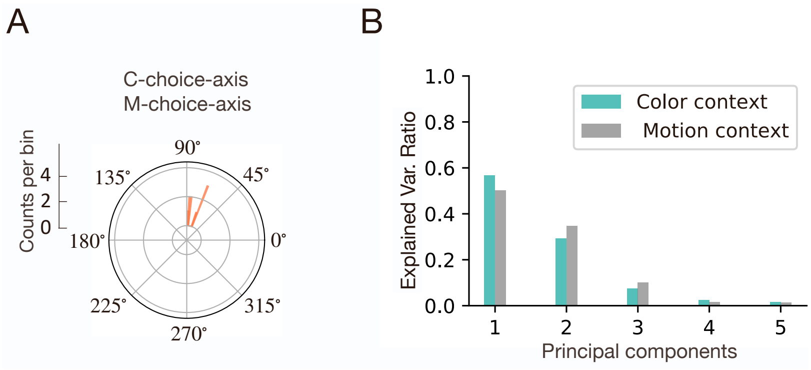

Figure S8: **(A)** Polar histogram of the angle between the `M-choice-axis` and `C-choice-axis`. **(B)** Ratio of explained variance of the first five PCs of subspace during the sensory stimulus epoch for the color and motion contexts, respectively.

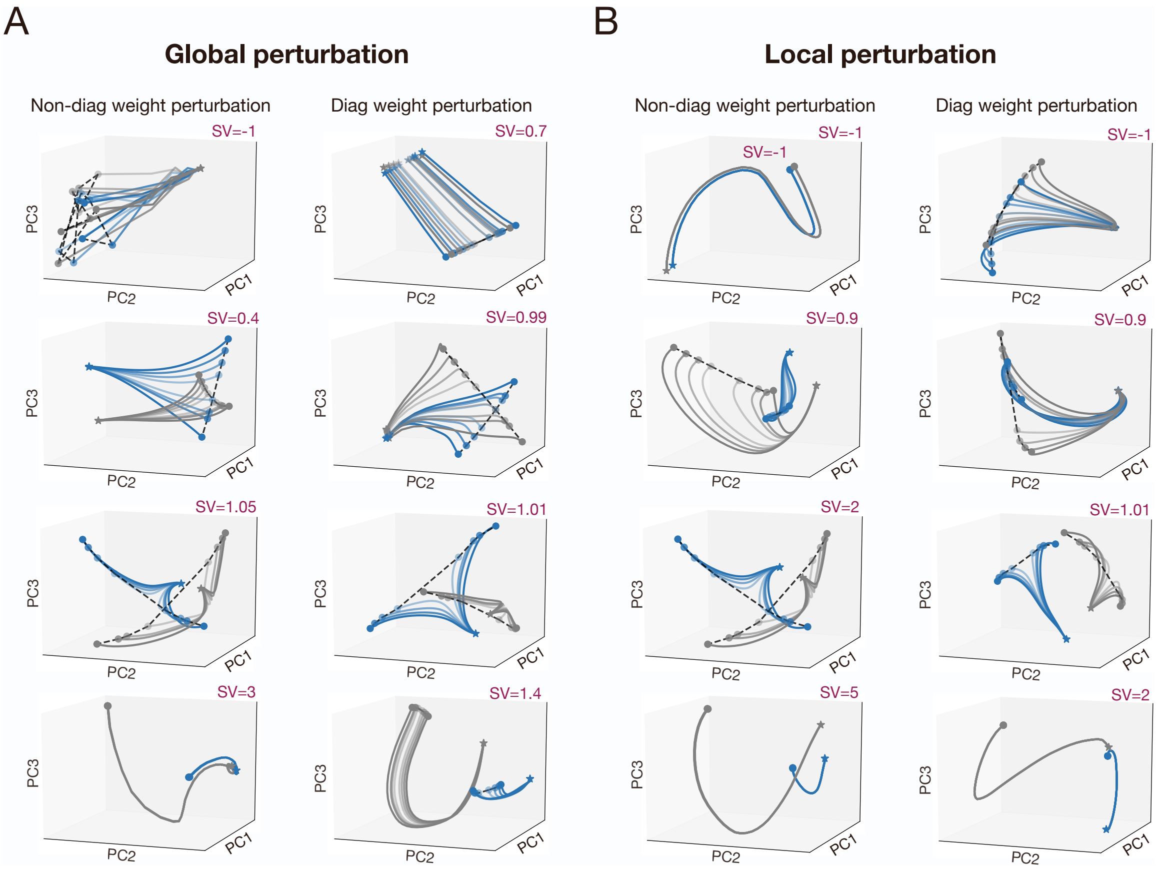

Figure S9: The neural trajectories under recurrent weight perturbation with different SV values. **(A)** Global recurrent weight perturbation was operated during the whole task period. The *left* column indicates non-diagonal weight perturbation, and the *right* column indicates diagonal weight. **(B)** Local perturbation was applied only locally in time during the delay epoch.

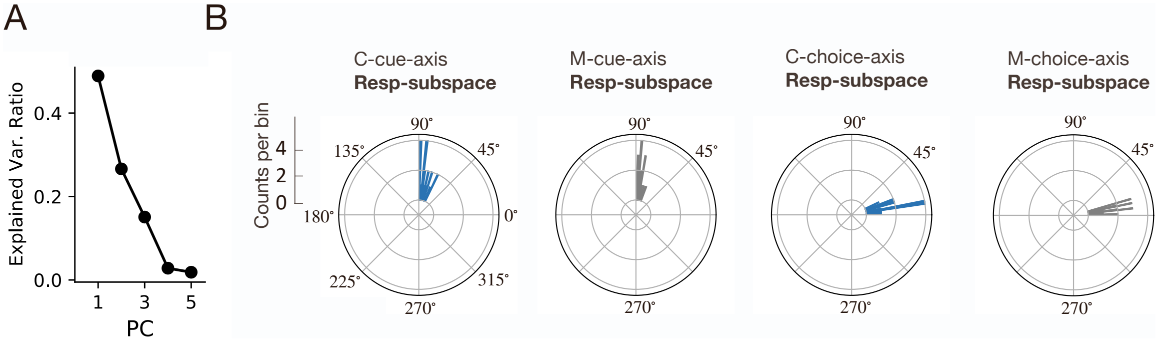

Figure S10: **(A)** Ratio of explained variance of the first five PCs of subspace during the response epoch. **(B)** The angle between four task-related axes and Resp-subspace.

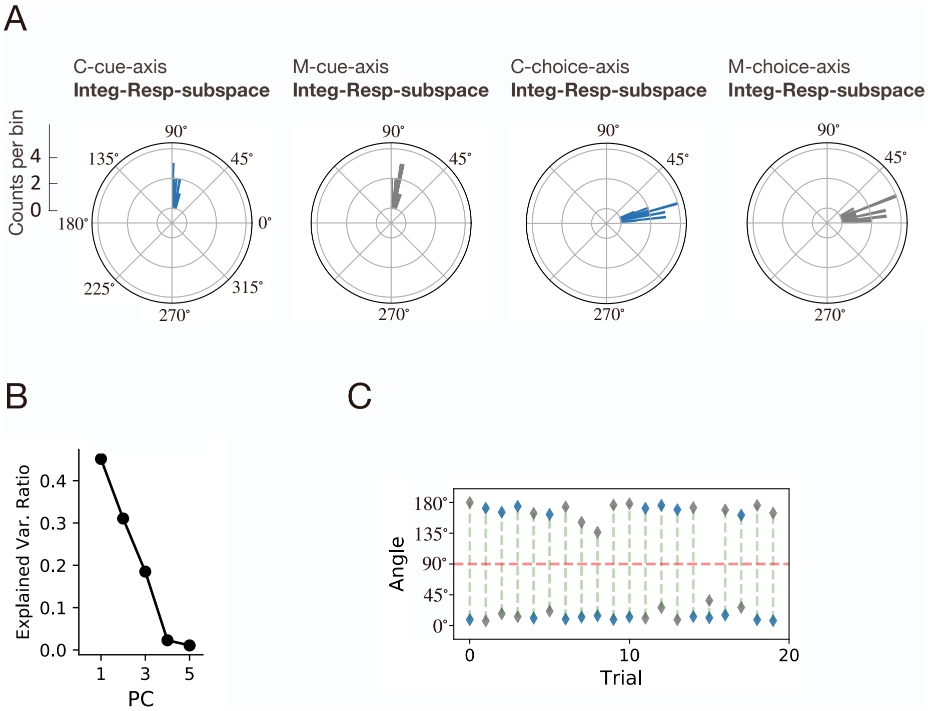

Figure S11: **(A)** The angle between four task-related axes and Integ-Resp-subspace. **(B)** Ratio of explained variance of the first five PCs of subspace. **(C)** The angle between the `C-cue-axis` (blue) or `M-cue-axis` (grey) and integ-resp-PC1 in 20 trials.
